# Linezolid-induced hematemesis, a rare and life-threatening adverse reaction. A case report of Karonga district in Malawi

**DOI:** 10.1093/omcr/omad020

**Published:** 2023-03-25

**Authors:** Samson Chitsulo, David Omotayo, Kuzani Mbendera, Frank Watson Sinyiza, Master Chisale, Balwani Chingatichifwe Mbakaya

**Affiliations:** Karonga District Hospital, Karonga, Malawi; Malawi National TB and Leprosy Elimination Program, Lilongwe, Malawi; Malawi National TB and Leprosy Elimination Program, Lilongwe, Malawi; Mzuzu Central Hospital, Mzuzu, Malawi; Mzuzu University, Mzuzu, Malawi; University of Livingstonia, Mzuzu, Malawi

## INTRODUCTION

The increase of Drug-Resistant Tuberculosis which includes Rifampicin-Resistant Tuberculosis (RR-TB), Multi Drug-Resistant Tuberculosis (MDR-TB) and Extensively Drug-Resistant Tuberculosis (XDR-TB) continues to be a major concern to National Tuberculosis Programs worldwide. RR-TB is TB caused by *Mycobacterium tuberculosis* strains that are resistant to rifampicin, whereas MDR TB is defined as TB with *in vitro* resistance to both isoniazid and rifampicin and XDR-TB are strains that fulfill the definition of MDR/RR TB and that are also resistant to any fluoroquinoles (Moxifloxacillin/Levofloxacillin) and at least one additional group A drugs i.e. Bedaquiline or Linezolid [1].

Previously, management of DR-TB has been a problem [2] but now with the coming of new and repurposed drugs, there is an improvement in the DR TB treatment outcomes and linezolid is among the best drug when added to Multi drug regimen and Extensive drug regimen with Bedaquiline and Pretomanid (BPaL). Linezolid is an antibiotic in the group of Oxazolidinones that inhibits bacterial protein synthesis. The toxicity profile of linezolid—which includes partially reversible myelosuppression, optic neuritis, neuropathy and lactic acidosis—limits its use. Studies have shown that treatment limiting toxicity can occur in as many as 11% of persons who receive treatment with linezolid. Adverse events mostly occur when linezolid is given at doses of >600 mg a day, but they can usually be identified early with routine monitoring and are often reversible upon discontinuation of the drug or lowering of the dose. Pyridoxine could be used when linezolid is administered to minimize side effects. Knowledge about the safety of linezolid during pregnancy and breastfeeding is limited so caution is advised and it is not clearly on the duration for side effects to appear [3].

## CASE PRESENTATION

**Figure 1:**
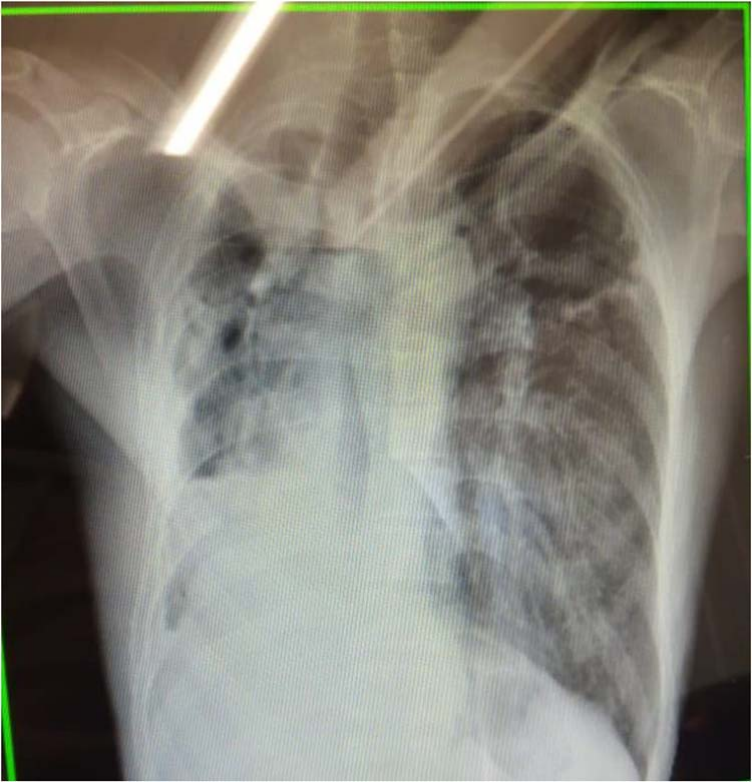
A chest X-ray which was taken before starting treatment with cavities more prominent to the left than right lung.

An 81-year-old HIV-negative Malawian man was diagnosed with Rifampicin resistance (RR) TB through Xpert MTB/Rif and was confirmed Multi Drug resistance (MDR) tuberculosis with culture and drug susceptibility testing which was done at National Reference Laboratory. He had no TB history even in his family, but there was history of working in mines in South Africa around 1970s. Contact investigation was done to his four household and close contacts and all were screened negative. Baseline investigations revealed hemoglobin of 12.4, platelets of 147, ALT of 6.8 and AST of 16.68 and chest X-ray with cavities on both lungs (Fig. 1). He had no comorbid condition and was not taking any other medicines. With the baseline assessment results, he was put on Longer Regimen All Oral comprised of Bedaquiline 400 mg for 2 weeks then 200 mg for 22 weeks making a total of 6 months, Linezolid 600 mg once a day, Clofazimine 100 mg once a day, Cycloserine 750 mg once a day, Levofloxacillin 1000 mg once a day and Pyridoxine 150 mg once a day. He was receiving this medication as an outpatient but under DOT by a trained nurse and a volunteer and was scheduled to be attending monthly clinical monitoring and follow-up review.

**Figure 2:**
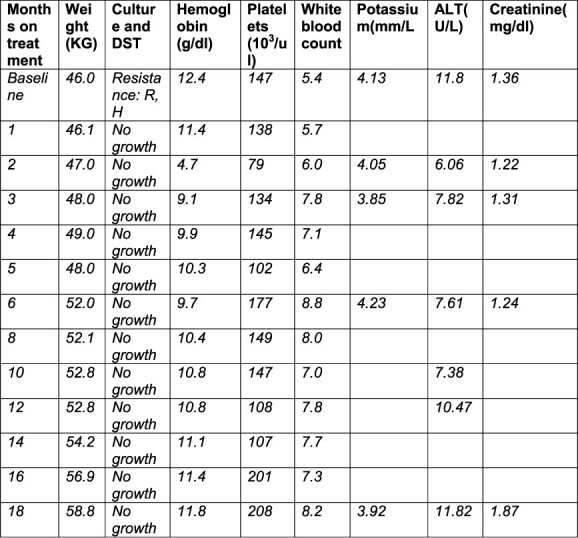
A table of baseline and monthly follow-up results.

At Week 8 while at home, he started vomiting fresh blood and was taken for admission at Karonga district hospital for intensive care and Rockall score was used to determine the severity of gastrointestinal bleeding (GI) bleeding although endoscopy was not done due to machine failure (Rockall score of 5). Upon assessment, there was no pertinent risk factor of upper GIT bleeding i.e. no history of smoking, alcohol intake, use of NSAIDs, anticoagulation, and there was no chronic liver disease and peptic ulcer disease. On examination, he had a low BP (85/50 mmHg), PR 118 b/min, RR 20 b/min, temperature of 36°C and the rest of the physical examination was normal. Whole blood count was done, hemoglobin of 4.7 from 12.4 and platelet count was 79 from 147 at baseline, respectively. He was transfused with four units of whole blood in total and linezolid was stopped immediately. All other medicines remained the same with no dose modification and the bleeding stopped spontaneously after 2 days while on treatment. He was diagnosed with an adverse event that was graded to 4 with as life-threatening. An adverse drug reaction report was filled and sent for pharmacovigilance at Malawi Pharmacy and Medicine Regulatory Authority. We had ruled out the possibility of other causes of vomiting blood like esophageal varices, esophageal tear, perforated peptic ulcer disease or esophagitis. There was nothing abnormal detected on ultra sound scan on the abdomen. He stayed in the ward for a week and then later was discharged with hemoglobin of 9.9 g/dl and platelet count of 145. He was to continue with monthly follow-up (Fig. 2), and iron tablets were supplied for a month. The patient culture converted to negative at Month 2 and he remained culture negative up until 18 months when he was finishing treatment. Linezolid was not re-introduced and there was no further episode of vomiting blood and patient was declared cured.

## DISCUSSION

Linezolid is an effective broad spectrum antibiotic; however, severe adverse effects including myelosuppression, optic neuritis, peripheral neuritis, lactic acidosis are common. Of note, it is the only medicine in Group A that has myelosuppression as one of the adverse event, whereas others in the same group like Bedaquiline, levofloxacin and Clofazimine mainly causesQT prolongation, headache, skin hyperpigmentation and hepatotoxicity. Some studies have reported that the rate of adverse events due to linezolid was high and the most adverse event reported frequently were myelosuppression, gastrointestinal reactions (diarrhea, vomiting, nausea), peripheral neuritis and optic neuritis and that the rate of myelosuppression including anemia and neutropenia was 32.9% higher and the presence of myelosuppression in the group with an initial dose of linezolid >600 mg per day was higher than that in the groups with an initial dose of linezolid less than or equal to 600 mg per day [4–6].

Linezolid toxicity is due to structural homology between target 23 s rRNA in *M. tuberculosis* and 16 s rRNA in human mitochondria resulting in a narrow therapeutic index and uncertainty around dose optimization. It is reported in one of the study that 38 XDR TB who treated with linezolid demonstrated the inverse association between linezolid trough concentrations associated with lower mitochondrial function and commented that long-term use of linezolid is associated with development of the mitochondrial toxicities of peripheral neuropathy, optic neuritis, myelosuppression and gastrointestinal reactions, which is contrary with this case report as it happened within 8 weeks of starting the treatment [7].

Other studies have reported that linezolid is effective and safe for the elderly with Gram-positive bacterial infection, but platelet count should be well monitored and that HIV may be a risk factor of linezolid-associated adverse events which in this case is HIV negative [8]. It is also reported that there is limitation on evaluation of the efficacy and safety of linezolid as efficacy in clinical practice is likely higher due to the additive or synergistic effect when used as combination with other drugs and that linezolid profile is not yet completely known and therefore need quality clinical research to better understand the appropriate dosage that clinicians can prescribe to minimize the probability of events [9, 10].

## CONCLUSION

Much as linezolid is given and presumed safe in patients on second-line anti-TB, knowledge about the safety of linezolid associated with hematemesis is limited. Published adverse event reports lack adequate information regarding hematemesis after initiation of linezolid. In our case, the relationship between linezolid and the onset of hematemesis is plausible. Health workers should be alert to the possibility of such adverse reaction.

## CONFLICT OF INTEREST STATEMENT

No conflict of interest.

## FUNDING

Malawi National TB/leprosy elimination program will support.
